# Parental marital conflict and internet gaming disorder among Chinese adolescents: The multiple mediating roles of deviant peer affiliation and teacher-student relationship

**DOI:** 10.1371/journal.pone.0280302

**Published:** 2023-01-17

**Authors:** Pinyi Wang, Xiong Gan, Hao Li, Xin Jin

**Affiliations:** 1 Department of Psychology, College of Education and Sports Sciences, Yangtze University, Jingzhou, China; 2 Department of Psychology, College of Education and Sports Sciences, Yangtze University College of Technology and Engineering, Jingzhou, China; North South University, BANGLADESH

## Abstract

A considerable amount of evidence suggests that parental marital conflict is an important factor in adolescents’ internet gaming disorder (IGD). However, the mechanism underlying this relationship remains unclear. Therefore, the purpose of this study is to explore the relationship between parental marital conflict and IGD among Chinese adolescents, and whether this relationship is mediated by deviant peer affiliation and teacher-student relationship. There were 698 Chinese adolescents that took part in the study (51.58% male; *M*_age_ = 13.52). They completed self-report questionnaires regarding perception of parental marital conflict, deviant peer affiliation, teacher-student relationship and IGD. Structural equation model (SEM) was used to examine the relationship between parental marital conflict and adolescents’ IGD, as well as the mediating roles of deviant peer affiliation and teacher-student relationship. Correlation analysis showed a positive correlation between parental marital conflict, deviant peer affiliation, and IGD, as well as a negative correlation between them and teacher-student relationship. The results of the SEM showed that parental marital conflict not only predicts adolescent IGD directly, but also through the mediation effects of deviant peer affiliation and teacher-student relationship. Additionally, deviant peer affiliation and teacher-student relationship not only play an independent but also a sequential mediating effect in the relationship between parental marital conflict and IGD. The relationship between parental marital conflict and IGD is mediated by deviant peer affiliation and teacher-student relationship, which has potential prevention and intervention value for adolescent IGD.

## 1. Introduction

With the rising popularity of internet, the issue of internet gaming disorder (IGD) among youths is becoming a worldwide problem [1]. According to the China Internet Network Information Center’s 48th Statistical Report, as of June 2021, the number of internet users in China had reached 1.011 billion, with 509 million online game users accounting for 50.4% of total internet users. 12.3% of internet users are between the ages of 10 and 19, according to the data [2]. IGD is defined as the physical, psychological, and social damage caused by the uncontrollable, excessive, and compulsive playing of internet games [3]. As IGD has become a global public health issue, it has been included in the updated version of the Diagnostic and Statistical Manual Disorders (DSM-5) and the International Classification of Diseases (ICD-11) [4, 5]. According to recent studies, Chinese adolescents have such a high prevalence of IGD, ranging from 2.97% to 13% [6, 7]. Researchers showed that IGD causes a variety of negative effects in adolescents, including anxiety, depression, insomnia, poor academic performance, and so on [8–11]. Therefore, research into the influencing factors and underlying mechanism of IGD among Chinese adolescents is necessary to create effective evidence-based interventions for the prevention of IGD in adolescents.

At present, research on IGD has gradually shifted from an initial focus on single ecological subsystem factors to a more comprehensive research orientation that incorporates multiple ecological subsystems such as family, school, and peer, which has deepened and improved understanding of IGD [7, 12–15]. The current study aims to investigate the family, school, and peers factors that impact IGD, and even the mechanism of occurrence, which can not only enrich relevant theories, but also have practical implications for the prevention and intervention of IGD in adolescents.

### 1. 1.Parental marital conflict and IGD

For adolescent, family is a vital place to grow up. Parental relationship, as a key component of the family system, has a significant impact on individual development [16, 17]. As children reach the age of adolescence, they become increasingly sensitive to their parents’ relationship, which has a direct impact on their development [18, 19]. Parental marital conflict is defined as verbal or physical aggressiveness between spouses as a result of a dispute or other factors [20]. According to attachment theory [21, 22], adolescents have trouble establishing safe attachments in families with high parental conflict levels. Adolescents who don’t build safe attachments are less able to handle stress and are more focused on having fun and getting rewards than having a long-term orientation. Therefore, they are more likely to be immersed in the virtual world through the internet while relieving bad emotions and develop internet gaming disorder [23]. Another aspect that could account for this association is the absence of parental network supervision [1, 14]. Adolescents are impressionable and interested as they go through a crucial phase of transition in their physical and mental development [24]. According to Ary et al., if the relationship between parents is terrible or there are too many conflicts, the norms of adolescent’s behaviors will be ignored, resulting in adolescents having difficulty controlling the time they spend on mobile phones and being prone to addiction and other problematic behaviors [25].

Previous researches had found that parental marital conflict is a significant predictor of adolescent IGD [1, 15, 26]. Tian et al. discovered, for example, that parental marital conflict had a significant positive predictive effect on adolescent IGD [1], suggesting that as parental marital conflict intensified, adolescent internet gaming disorder behavior increased significantly. Despite the fact that the vast majority of studies have confirmed a positive relationship between parental marital conflict and IGD, some researchers disagree with that statement [27, 28]. According to the self-reported results of adolescents, researchers proposed that there is only a weak correlation between parental marital conflict and IGD of adolescents, and that there is no correlation after controlling for gender and gaming type [29]. As a matter of fact, as individuals grow and spend more time in school, teachers [30] and peers [31] get to be increasingly important. As a result, family factors such as parental marital conflict may be a distant predictor of IGD throughout adolescence. Therefore, it is critical to investigate the possible mechanism of the relationship between adolescent parental marital conflict and IGD in order to obtain a better understanding of the relationship between them.

### 1.2. Deviant peer affiliation as a mediator

Deviant peer affiliation is the socialization of peers who engage in illegal or socially unacceptable behavior [32]. On the one hand, as described in social learning theory [33], adolescents develop IGD by learning and imitating the behavior of deviant peers [13]. Furthermore, the norms that develop among deviant peers promote the occurrence and persistence of adolescent delinquent behaviors. To gain acceptance and approval from the peer group, adolescents further reinforce their problem behaviors [14]. Ample studies have found that deviant peer affiliation can significantly predict IGD problems in adolescents [23, 34, 35]. On the other hand, adolescents who are unable to meet basic psychological needs in parent-child interaction may be maladjusted and more likely to seek support from peers [36, 37], as per the stage-environment fit perspective [38]. For adolescents in families with high levels of parental conflict, making friends with deviant peers is more likely to attract the attention of their parents to compensate for parental neglect due to the conflict [23]. Parental marital conflict has been shown in studies to have a positive predictive effect on adolescents’ deviant peer affiliation [13, 26]. According to biological ecology theory [39], adolescent development can be influenced by multiple ecological systems, such as family, school, and peers, and all ecological subsystems are interconnected. Among the various subsystems that affect adolescent physical and mental development, risk factors in one subsystem increase adolescents’ exposure to risk factors in another subsystem and lead to adolescent problem behavior [37]. Peer interaction also has a more immediate impact on adolescents as they spend more time in school. Therefore, deviant peer affiliation may have a proximal effect on IGD as a mechanism of the relationship between family factors and IGD.

Through a questionnaire survey, Bao et al. demonstrated that deviant peer affiliation played a completely mediating role in the relationship between parent-child relationship and IGD [13]. Other researchers have found that parental marital conflict influences adolescent IGD via deviant peer affiliation [1, 26]. As a result, based on the above mentioned theories and related research, this study will explore the role of deviant peer affiliation as a mediator in the relationship between parental marital conflict and IGD.

### 1.3. Teacher-student relationship as a mediator

The teacher-student relationship is the psychological relationship that exists between teachers and students in the school context, and it primarily consists of emotional, cognitive, and behavioral communication [40]. For adolescent in school, teacher-student relationship is an important form of social communication. On the one hand, a positive teacher-student relationship can provide adolescents with social support in learning and problem solving. on the other hand, according to attachment theory [21, 22], when individuals do not obtain warmth from important others (such as teachers), they feel insecure and have difficulty meeting their inner needs, which may lead them to seek other sources, such as the internet, to compensate for the emotional hunger they cannot satisfy in the real world, increasing the likelihood of internet gaming disorder. According to empirical research, teacher-student relationship is closely related to adolescent anxiety and depression [30]. Researchers also discovered that teacher-student relationship is an important factor in adolescent IGD, and that a positive teacher-student relationship protects adolescent from developing a disorder to internet games [41]. Furthermore, previous research showed that parental marital conflict leads to a negative attitude and apathy in future interpersonal communication among teenagers [12], resulting in a negative teacher-student relationship and an increase in problem behavior (e.g. bullying) [42]. As an important part of the school subsystem, teacher-student relationships can also act in concert with the family subsystem on adolescent behavioral outcomes. Previous studies have shown that in the relationship between parental corporal punishment and teenagers’ IGD, the school connection serves as an intermediary [41]. This suggests that teacher-student relationship may be a connection between family factors and IGD.

### 1.4. The relationship between deviant peer affiliation and teacher-student relationship

The relationship between deviant peer affiliation and teacher-student relationship was also investigated by some researchers. The results show a significant negative correlation between deviant peer affiliation and teacher-student relationship, with teenagers who make deviant companions showing less class participation, which then affects the teacher-student relationship [41]. According to Mercer and Derosier [43], teachers will form corresponding cognitive schemata based on the status and characteristics of students in the class, and will communicate with students in ways that reflect their cognitive schemata. Related study has found that the higher the level of deviant peer affiliation, the worse the teacher-student relationship is [44]. Adolescents spend more time with their classmates or at school as they grow older, and the impact of peers and teachers becomes more essential. Given the results of previous studies, deviant peer affiliation can have an impact on an individual’s teacher-student relationship. Thus parental marital conflict, while influencing IGD through deviant peer affiliation, is likely to contribute to IGD by worsening teacher-student relationships based on the influence of deviant peer affiliation. Despite the fact that both deviant peer affiliation [1, 26] and teacher-student relationship [42] have been demonstrated to be mediators of the parental effect in research, few studies have looked at their mediating functions in the same model. As a result, our study compensates for this weakness by examining the mediating effects of the combined.

### 1.5. The present study

**Fig 1:**
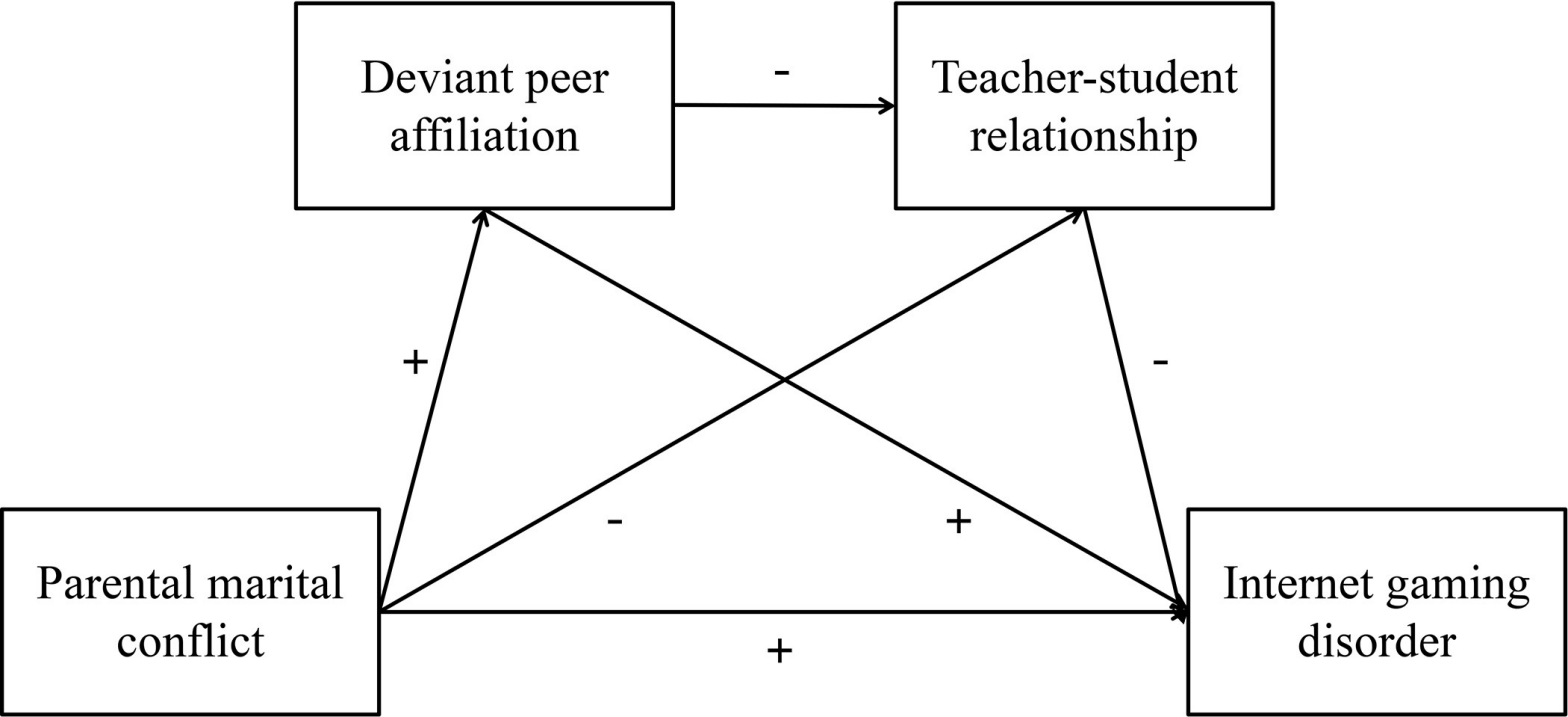
Proposed mechanism of the association between parental marital conflict and IGD.

We aimed to examine the influence of adolescents’ family, peers, and school factors on IGD and its internal mechanism in the same model, based on the above mentioned literature review and related theories. We believe that parental marital conflict is a distal predictor of IGD, meaning that it can significantly predict IGD in adolescents (hypothesis 1). The connection between parental marital conflict and IGD is mediated by deviant peer affiliation (hypothesis 2) and teacher-student relationship (hypothesis 3). Deviant peer affiliation can have a negative impact on the teacher-student relationship, implying that deviant peer affiliation and the teacher-student relationship play multiple mediating roles in the association between parental marital conflict and IGD (hypothesis 4). The whole proposed model is depicted in Fig 1. Taken together, the two mediators of deviant peer affiliation and the teacher-student relationship are expected to shed light on the relationship between parental marital conflict and adolescent IGD. Identifying the mechanism by which the parental marital conflict is linked to IGD has crucial implications for theory and prevention, and is a step closer to developing an integrative framework to understand the complex relationships among variables in our study.

## 2. Methods

### 2.1. Participants

Students from the first and second grades of two junior middle schools in Hubei province from China, were chosen as research participants using a simple random cluster sampling method. A total of 740 questionnaires were sent out, and 698 were successfully received, and the effective recovery was 94.3%. 23.5% of those who took part were from cities, while 76.5% were from rural areas. There are 360 boys (51.58%) and 338 girls (48.42%). The participants ranged in age from 12 to 16, with an average age of 13.52 years (*SD* = 0.70).

### 2.2. Procedures

The study was approved by the Research Ethics Committee of the College of Education and Sports Sciences, Yangtze University. A group test was conducted in the classroom, with graduate students trained in professional psychology acting as the conductor. With the informed consent of students and their parents or legal guardians involved, an anonymous questionnaire survey was conducted. The questionnaire of the students who participated voluntarily was collected after they finished, while the remainder were not required to. All participants were told that their answers will be kept confidential and they can quit at any time if they feel uncomfortable and were required to complete all items independently throughout the process.

### 2.3. Measures

#### 2.3.1. Parental marital conflict

The Children’s Perception of Interparental Conflict Scale (CPIC) revised by Chi and Xin [20] was used, which contains seven dimensions. According to Yu et al. [11], this study uses three dimensions of conflict frequency, conflict intensity, and conflict resolution to assess the level of marital conflict between parents. This scale contains nineteen items, such as “parents frequently dispute or disagree with each other”. All items were rated on a 4-point Likert-type scale ranging from 1 (very inconsistent) to 4 (very consistent). Each dimension’s average score is calculated (after reverse-coding when necessary), with higher sores indicating higher levels of parental marital conflict. This measure has demonstrated good reliability and validity among Chinese adolescents [45, 46]. In the present study, the Cronbach’s alphas coefficients for the three subscales and the overall scale were 0.71, 0.80, 0.80, and 0.90 respectively.

#### 2.3.2. Deviant peer affiliation

Participants were asked to write how many of their close friends had engaged in deviant behaviors (e.g. fighting, cheating, truancy, and smoking) in the previous six months using the Deviant Peer Affiliation Questionnaire (DPA), a 12-items questionnaire revised by Zhu et al. [13]. All items were rated on a 5-point scale ranging from 1 (none) to 5 (more than or equal to 6). The mean score of all items was taken, with higher score suggesting more deviant peer affiliation. This measure has demonstrated good reliability and validity among Chinese adolescents [47]. The Cronbach’s alphas coefficient for the overall questionnaire was 0.89 in this study.

#### 2.3.3. Teacher-student relationship

The questionnaire of teacher-student relationship compiled by Jia et al. [48] was used. The questionnaire contains seven items and all items were rated on a 4-point scale ranging from 1 (never) to 4 (always). Item scores were averaged to create a composite of teacher-student relationship, with higher scores indicating higher levels of teacher-student relationship. This measure has demonstrated good reliability and validity among Chinese adolescents [49]. In the current study, the questionnaire’s Cronbach’s alphas coefficient was 0.81.

#### 2.3.4. Internet gaming disorder

Internet gaming disorder was measured with eleven items revised by Yu et al. [49]. All items were rated on a 3-point scale ranging from 0 (never) to 2 (frequently). The average score of all items was calculated, with higher scores indicating bigger likelihood of developing a disorder to internet games. This measure has demonstrated good reliability and validity among Chinese adolescents [11]. The questionnaire’s Cronbach’s alphas coefficient was 0.82 in this study.

### 2.4. Statistical analysis

For descriptive statistics and correlation analysis, SPSS24.0 was utilized. Traditional statistical analysis methods cannot properly handle latent variables and cannot properly evaluate the relationships of latent variables [50]. We used structural equation modeling (SEM) to examine mediation effects with Mplus Version 7.4. We used bootstrapping with 2000 iterations to test the statistical significance of the paths in each model. We included five fit indices to assess the adequacy of models in the current study: the ratio of chi-square over degrees of freedom (*χ*^*2*^/*df*), comparative fit index (*CFI*), Tucker-Lewis index (*TLI*), root mean square error of approximation (*RMSEA*), and standardized root mean square residual (*SRMR*). Following their cut-off scores [51], model fit is good when *χ*^*2*^/*df* ≤ 3; *CFI* ≥ 0.95, *TLI* ≥ 0.95; *RMSEA* ≤ 0.06, and *SRMR* ≤ 0.08.

## 3. Results

### 3.1. Preliminary analyses

Because the data were collected through self-reporting, there may be a common method bias. Some approaches, such as reverse scoring and anonymous filling, were used to control the process in order to decrease the influence of the common method bias. Harman single factor test was used to inspect this bias [52]. The results reveal that the eigenvalues of ten factors are more than 1, and the variance explained by the first factor is 18.69%, which is much less than the critical value of 40%, indicating that there is no significant common method bias in this study.

Means, standard deviations, skewness, kurtosis, and correlations are displayed in Table 1, where the skewness and kurtosis coefficients are all smaller than 3 and 8, respectively, in absolute terms [53]. Consequently, the information complies with the specifications of a roughly normal distribution. Both parental marital conflict and deviant peer affiliation are positively correlated with IGD (*r*_*1*_ = .23, *p* < .01; *r*_*2*_ = .34, *p* < .01). Teacher-student relationship was negatively correlated with IGD(*r* = -.20, *p* < .01). In addition, the significant correlations appeared among parental marital conflict, deviant peer affiliation and teacher-student relationship.

**Table 1:** Descriptive statistics and correlations for all variables.

Variables	*M*	*SD*	*Skewness*	*Kurtosis*	1	2	3	4
1.Parental marital conflict	2.13	0.53	0.08	-0.15	1			
2.Deviant peer affiliation	1.68	0.73	1.83	3.97	0.22**	1		
3.Teacher-student relationship	2.87	0.66	0.69	1.91	-0.26**	-0.22**	1	
4.Internet gaming disorder	0.22	0.18	1.04	0.94	0.23**	0.34**	-0.20**	1

Note

***p < .001

**p < .01

*p < .05

the same below.

### 3.2. The multiple mediating roles of deviant peer affiliation and teacher-student relationship

The multiple mediating effects were tested using a latent variable structural equation model with parental marital conflict as the independent variable, IGD as the dependent variable, deviant peer affiliation and teacher-student relationship as the mediating variables. According to Wu and Wen [54], the balance method in the factorial algorithm was used as the main method for one-dimensional scales project packaging of deviant peer affiliation, teacher-student relationship, and internet gaming disorder. The items under each scale were packed into three groups with comparable loadings and variance using the loadings of the items as the foundation to maintain the model’s stability and reliability of data fitting, and reduce data bias. For the Children’s Perception of Interparental Conflict Scale, it is a common method to package the items of the same dimension directly in a group. Therefore, the three dimensions of this Scale were utilized. The measurement model now has four latent variables and twelve observed variables. The parameters of the measurement model are estimated and tested using the maximum likelihood method of the covariance structure model, and the fitting index is as follows: *χ*^*2*^/*df* = 2.23, *CFI* = 0.98, *TLI* = 0.98, *RMSEA* = 0.04, *SRMR* = 0.05. The model fits well and demonstrates that these indexes can effectively estimate all latent variables.

**Fig 2:**
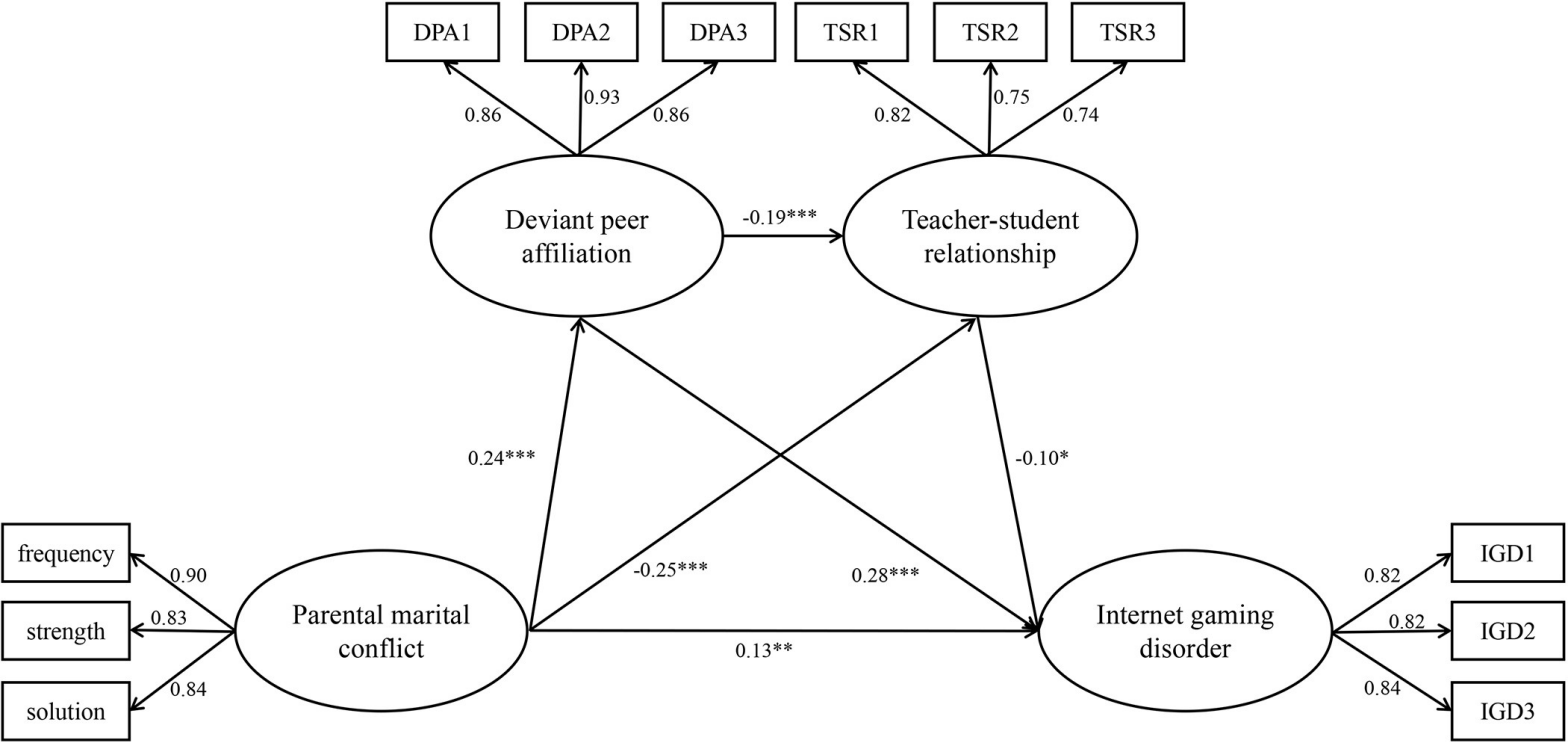
Proposed mechanism of the association between parental marital conflict and IGD.

Firstly, we examine the direct effect of parental marital conflict on adolescents’ IGD, using parental marital conflict as a predictor and IGD as a result variable. The results indicate that the model is well-fitting: *χ*^*2*^/*df* = 1.39, *CFI* = 0.99, *TLI* = 0.99, *RMSEA* = 0.02, *SRMR* = 0.02. The path coefficient of parental marital conflict to IGD is significant (*β* = .27, *t* = 6.56, *p* < .001), and hypothesis 1 is proved. The intermediary variables of deviant peer affiliation and teacher-student relationship are then added to the model to evaluate the multiple intermediary effects, as per the research hypothesis. The structural equation model’s fitting indexes are as follows: *χ*^*2*^/*df* = 2.80, *CFI* = 0.98, *TLI* = 0.97, *RMSEA* = 0.05, *SRMR* = 0.06. Fig 2 depicts the structural equation model: parental marital conflict predicts IGD positively (*β* = .13, *t* = 3.01, *p* < .01), parental marital conflict positively predicts deviant peer affiliation (*β* = .24, *t* = 6.02, *p* < .001), and deviant peer affiliation positively predicts IGD (*β* = .28, *t* = 7.05, *p* < .001), proving hypothesis 2. Parental marital conflict predicts teacher-student relationship negatively (*β* = -.25, *t* = -5.91, *p* < .001), and teacher-student relationship negatively predicts IGD (*β* = -.10, *t* = -2.16, *p* < .05), proving hypothesis 3. Therefore, deviant peer affiliation and teacher-student relationship have a parallel effect in moderating parental marital conflict and IGD.

We use the bias correction Bootstrap method by Fang et al. [55] to test the significance of the effect, and 2000 samples are setup to be extracted in Mplus version 7.4. If the parameter estimation value is significant, the 95% confidence interval of the bootstrap does not contain 0. If not, it means that the parameter estimation value isn’t significant [56]. Table 2 presents the results: the 95% confidence intervals for “PMC→DPA→IGD” and “PMC→TSR→IGD” don’t contain 0, indicating that the two intermediate paths are significant and the mediation effect values are 0.067 and 0.024 respectively, accounted for 70.53% and 25.26% respectively. The 95% confidence intervals for “PMC→DPA→TSR→IGD” don’t contain 0, with a mediating effect value of 0.004, accounted for 4.21%. Our findings suggest that deviant peer affiliation and teacher-student relationships play a sequential mediating role between parental marital conflict and IGD, proving hypothesis 4.

**Table 2:** Standardized estimates and 95% CIs for direct and indirect effects.

Path	Standardized Estimate	95% *CI*
Direct effect		
PMC→IGD	0.128	[0.046, 0.203]
Total indirect effect		
PMC→IGD	0.095	[0.059, 0.143]
Specific indirect effect		
PMC→DPA→IGD	0.067	[0.038, 0.108]
PMC→TSR→IGD	0.024	[0.003, 0.053]
PMC→DPA→TSR→IGD	0.004	[0.001, 0.012]

Note: PMC = parental marital conflict, DPA = deviant peer affiliation, TSR = teacher-student relationship, IGD = internet gaming disorder.

## 4. Discussion

The current researchers focused on the impact of environmental factors on IGD in adolescence. Some results have been obtained about the relationship between parental marital conflict and adolescent IGD [1, 15, 26]. However, the underlying mediating mechanisms need to be explored more thoroughly. This study contributed to growing of studies by building and testing a model that included deviant peer affiliation and teacher-student relationship as important mediators of the connection between parental marital conflict and IGD, examining the effect of different ecological subsystems on IGD and its mechanisms, which has crucial implications for theory and prevention.

### 4.1. Parental marital conflict and IGD

The findings demonstrate that the higher the level of parental marital conflict, the higher the adolescents’ IGD level, which is in accordance with previous findings [1, 15, 26]. Living in a long-term family environment marked by parental conflict will result in unpleasant feelings and a lack of ability to handle problems later [57, 58]. Conflicts between parents will also cause adolescents’ inner needs to be unsatisfied, leading them to turn to online games to fill in this gap [24, 59], such that the likelihood of internet gaming disorder is increased. Additionally, parents often lack the time and material resources to offer their teenagers balanced leisure options since disagreement requires too much time and energy from both parties. As a result, adolescents living in families with high levels of parental marital conflict spend more energy on online games compared to families with harmonious parental relationships, which also increases their risk of internet gaming disorder [24]. These findings lay the foundation for more research into the mediating roles of deviant peer affiliation and teacher-student relationship in the connection between parental marital conflict and IGD. The structural equation model’s results show that deviant peer affiliation and teacher-student relationship both play parallel mediation roles between parental marital conflict and IGD. As a result, parental marital conflict can affect adolescents’ IGD not only directly, but also indirectly through deviant peer affiliation and teacher-student relationship. Meanwhile, deviant peer affiliation predicted teacher-student relationship significantly, showing a sequential mediation mechanism, and hypothesis 4 was confirmed.

### 4.2. The multiple mediating roles of deviant peer affiliation and teacher-student relationship

The mediating effect of deviant peer affiliation is consistent with previous researches [1, 13, 14, 26]. Parental marital conflict can predict deviant peer affiliation [36], and deviant peer affiliation can predict adolescent IGD in a significant way [23, 34, 35]. According to the emotional security theory [19], parental marital conflict diminishes adolescents’ sense of security, which can be alleviated by deviant peer support. In addition, high levels of parental conflict in the marriage led to higher levels of feelings of uncontrollability among adolescents. Delinquent peers, due to seemingly higher levels of autonomy, tend to be the primary choice of adolescents to interact with in order to seek needs satisfaction [14]. Adolescents develop IGD by observing and imitating the abnormal actions of their peers. And the norms that develop among deviant peer groups can reinforce their problem behaviors [14]. Therefore, adolescent who live in families with a lot of parental conflicts are more likely to associate with deviant peers to satisfy their security requirements, and develop IGD because of imitating and peer pressure. This finding also confirms the notion that different subsystems are interconnected rather than developing independently. In the family subsystem, the risk factor of parental marital conflict influences deviant peer interactions in the peer subsystem. The risk factors in the various subsystems interact to affect adolescent’s behavioral outcomes. In addition, the study found that teacher-student relationship plays an intermediary effect between parental marital conflict and IGD, which is consistent with the results of related studies [42, 60]. Parental conflict in the family system, according to Yeung et al., is a form of mental domestic violence for children that has long-term impacts on children and affects teacher-student relationships or closeness after they receive an education [61]. Therefore, we believe that the higher the level of marital conflict between parents, the worse the relationship between teachers and students. A positive teacher-student relationship is critical to a student’s development, whereas a negative teacher-student relationship causes students to feel unsafe and turn to other environments (e.g. internet games) to meet their psychological requirements [60]. Adolescents with poor teacher-student relationships, according to Denny et al., will lose their connection with school, increasing the risk of IGD and other problematic behaviors [62]. Teachers play a crucial role in the educational life of adolescents, and positive teacher-student relationships encourage students to actively engage in coursework and learning activity, which reduces the likelihood that they would engage in internet gaming activities [30]. Therefore, parental marital conflict also influences adolescent IGD by worsen teacher-student relationship, consistent with the biological ecology theory [39].

Furthermore, the present study also found that deviant peer affiliation and teacher-student relationship play sequential mediation roles between parental marital conflict and adolescent IGD. In other words, adolescents who experience a high level of parental marital conflict are more likely to have plentiful deviant peers, which may contribute to the deteriorate of teacher-student relationship and in turn, result in higher likelihood of IGD. This finding may be due to that teacher-student relationship can be affected by adolescent deviant peer affiliation, which could help researchers better understand the link’s underlying mechanisms. That is, when students interact with more delinquent peers, the teacher-student relationship deteriorates due to the adolescent’s violation of school norms and inability to complete academic tasks as required [41, 44]. Fewer studies have previously noted this, but the link between deviant peer affiliation and teacher-student relationship deserves our attention. From the teacher’s perspective, reducing the impact of deviant peer affiliation on the teacher-student relationship and avoiding the pernicious effects of deviant peer interactions are crucial to individual development.

It’s worth noting that even mediation variables are taken into account, the straight path remains significant. This finding suggests that various subsystems, such as family, peers, and school, have a significant effect on individual behavior throughout adolescence, which is in line with the multiple attachment theory [42] and earlier research [7, 26, 34, 41]. However, some researchers believe that when the individual growing environment changes, the influence of family as a distant factor on adolescents decreases, and IGD is mostly driven by other factors, which contradicts the findings of this study [13]. Therefore, more research into how family factors influence adolescents’ IGD behavior is required. From a practical standpoint, these findings highlight the importance of clarifying such processes in order to improve prevention and intervention that has the ability to alter at various stages.

### 4.3. Contributions and limitations

There are some important contributions of this study. First, the findings extend our insights into the underlying mechanism of this relationship by demonstrating that in addition to the mediators discovered in previous research (e.g. anxiety, depression, sensation seeking), deviant peer affiliation and teacher-student relationship can play important mediating roles in the links between parental marital conflict and IGD. Furthermore, by including both mediators in the model, the current study examined the independent and sequential mediating effects of these mediators. Third, these findings may provide practical implications for how to prevent and intervene adolescents’ IGD. For instance, parents should try to avoid conflict in front of their children to keep a harmonious family environment. In addition, teachers should do a good job of instruction, help adolescents reduce bad social contact, and actively build a good teacher-student relationship.

Despite the above-mentioned contributions, some limitations of this study should be mentioned. First, this study uses cross-sectional design to explore the casual relationship between variables, and the causality relationships can’t be delineated. Longitudinal design is expected to be used in the future to better explore the causal relationship between variables. Second, we used the self-reporting method to collect data, which may be affected by social desirability. Future research should use multiple methods and multiple informants to increase the validity of the findings. Third, we wanted to reveal the causes of IGD in adolescents by investigating its propensity through the Internet Gaming Disorder Scale. However, it remains unknown whether individuals who score high on the scale will be diagnosed with IGD in clinical diagnosis. Therefore, future exploration of the antecedents of IGD should be combined with clinical indicators. Fourth, this study found that the relationship between parental marital conflict and IGD was partially mediated, suggesting that a further understanding of this relationship requires information on other mediators. Finally, the current study was conducted with a sample of Chinese adolescents, and future research should include cross-cultural samples to test the generalizability of our findings.

## 5. Conclusion

Based on the biological ecology theory and attachment theory, this study explored the influence of family, school and peer subsystems on individual IGD and their internal potential mechanisms. This study explores the multiple mediating roles of deviant peer affiliation and teacher-student relationship in parental marital conflict and adolescent IGD, and draws the following conclusions:

1. Parental marital conflict could positively predict adolescent IGD;
2. The relationship between parental marital conflict and IGD is mediated by deviant peer affiliation;
3. The relationship between parental marital conflict and IGD is mediated by teacher-student relationship;
4. Deviant peer affiliation and teacher-student relationship play a role of chain intermediary between parental marital conflict and IGD.

By exploring the relationships and underlying mechanism, we can see that as individuals develop and spend more time in school, teachers and peers in the school environment play a more direct role in individual behavior as proximal factors. At the same time, however, family systems still have an impact that cannot be ignored. Thus, these subsystems jointly act on individual behavioral outcomes, and interventions for individual maladaptive behaviors need to start from different subsystems.

## Supporting information

S1 Dataset Dataset used for analyses in present study. (RAR)
